# Intestinal 3-methylindole exacerbates sepsis-induced neuroinflammation through microglial NLRP3 inflammasome activation

**DOI:** 10.1128/spectrum.01542-26

**Published:** 2026-10-06

**Authors:** Heng Fang, Simin Wu, Peixian Huang, Qiuping Zhou, Ying Chen, Yiyan Lin, Chunbo Chen, Yiyu Deng

**Affiliations:** 1 Department of Critical Care Medicine, Guangdong Provincial People's Hospital, Guangdong Academy of Medical Sciences, Southern Medical University 70570, Guangzhou, China; 2 Department of Emergency, Shenzhen People's Hospital, The Second Clinical Medical College of Jinan University, The First Affiliated Hospital of Southern University of Science and Technology 12387, Shenzhen, China; University of Arkansas for Medical Sciences, Little Rock, Arkansas, USA

**Keywords:** gut microbiota, *Escherichia coli*, septic neuroinflammation, 3-methylindole, NLRP3

## Abstract

Gut microbiota regulates neuroinflammatory responses involved in the pathogenesis of sepsis-associated encephalopathy (SAE). However, the underlying mechanism remains elusive. 16S rRNA sequencing of fecal samples revealed an expansion of the *Enterobacteriaceae* family in septic mice, and quantitative PCR further detected an increase in gut *Escherichia coli*. Subsequent bacterial transplantation experiments showed that the exacerbation of neuroinflammatory responses in sepsis by gut microbiota dysbiosis is due to the expansion of gut *E. coli*. Furthermore, we observed an increase in *E. coli*-derived 3-methylindole (3MI) in septic mice. Although 3MI alone does not directly induce neuroinflammation, it synergistically exacerbates neuroinflammatory responses with lipopolysaccharide. Mechanistically, 3MI may reduce the ubiquitination level of NLRP3 and enhance NLRP3 inflammasome activation in lipopolysaccharide-stimulated microglia. These findings suggest that *E. coli*-derived 3MI is a contributing factor in aggravating neuroinflammation in SAE, offering new insights into therapeutic strategies for SAE.

**IMPORTANCE:**

The gut-brain axis is increasingly implicated in sepsis‑associated encephalopathy (SAE) pathogenesis, yet the precise microbial mechanisms driving neuroinflammation remain poorly defined. This study identifies a specific gut microbial pathway and reveals that sepsis-induced expansion of *Escherichia coli* and its overproduction of the metabolite 3-methylindole are key aggravating factors. Notably, while 3-methylindole alone is non-inflammatory, it acts synergistically with lipopolysaccharide to drive NLRP3 inflammasome activation in microglia, likely via reducing NLRP3 ubiquitination. These findings pinpoint a gut bacterium-derived metabolite as a critical amplifier of brain inflammation in sepsis, which contributes to a new mechanistic understanding of SAE pathogenesis.

## INTRODUCTION

Sepsis-associated encephalopathy (SAE) is a common and severe central nervous system complication in the intensive care unit (ICU), characterized by diffuse brain dysfunction (1, 2). Patients with SAE exhibit high ICU mortality rates, and survivors often suffer from long-term cognitive impairment (3). Despite continuous improvements in sepsis management, the precise pathogenesis of SAE remains incompletely understood, and specific therapeutic interventions are still lacking (4–6). Therefore, in-depth investigation into its pathophysiological mechanisms and identification of key intervention targets are urgently needed.

The “microbiota-gut-brain axis” theory may represent a promising avenue for the intervention of SAE (7, 8). The gut microbiota, a complex microbial community within the intestines, normally exists in a dynamic balance with the host (9, 10). During sepsis, this balance is disrupted, leading to dysbiosis characterized by compositional alterations (e.g., expansion of opportunistic pathogens, such as *Enterobacteriaceae*) and metabolic dysfunction, with abnormal accumulation of metabolites, including hippuric acid (11–13). Research indicates that gut microbiota-derived metabolites, such as short-chain fatty acids and tryptophan metabolites, are involved in the pathogenesis of Alzheimer’s disease and Parkinson’s disease by modulating microglial activity and neuroinflammation (14, 15). Our earlier work revealed that the expansion of the intestinal *Enterobacteriaceae* is associated with increased susceptibility to SAE in septic mice (16); however, the underlying mechanisms remain to be further elucidated.

The core pathological mechanism of SAE is closely associated with infection-induced excessive neuroinflammation (17, 18). During this process, microglia, as the innate immune cells of the central nervous system, play a critical role in driving neuroinflammation through their sustained aberrant activation (19, 20). Notably, gut microbiota metabolites, serving as messengers in gut-brain communication, introduce an additional regulatory element in microglial activation (21). Specific metabolites, such as short-chain fatty acids, are being explored for their potential to mitigate neuroinflammation (22). Based on these findings, we hypothesized that certain abnormally accumulated gut-derived metabolites may cross the impaired blood-brain barrier and contribute to the regulation of microglial inflammatory responses under septic conditions. Here, we identified the specific molecules derived from gut dysbiosis that regulate neuroinflammation in SAE and investigated the underlying mechanisms.

## RESULTS

### Gut microbiota dysbiosis characterized by *E. coli* expansion in septic mice

**Fig 1:**
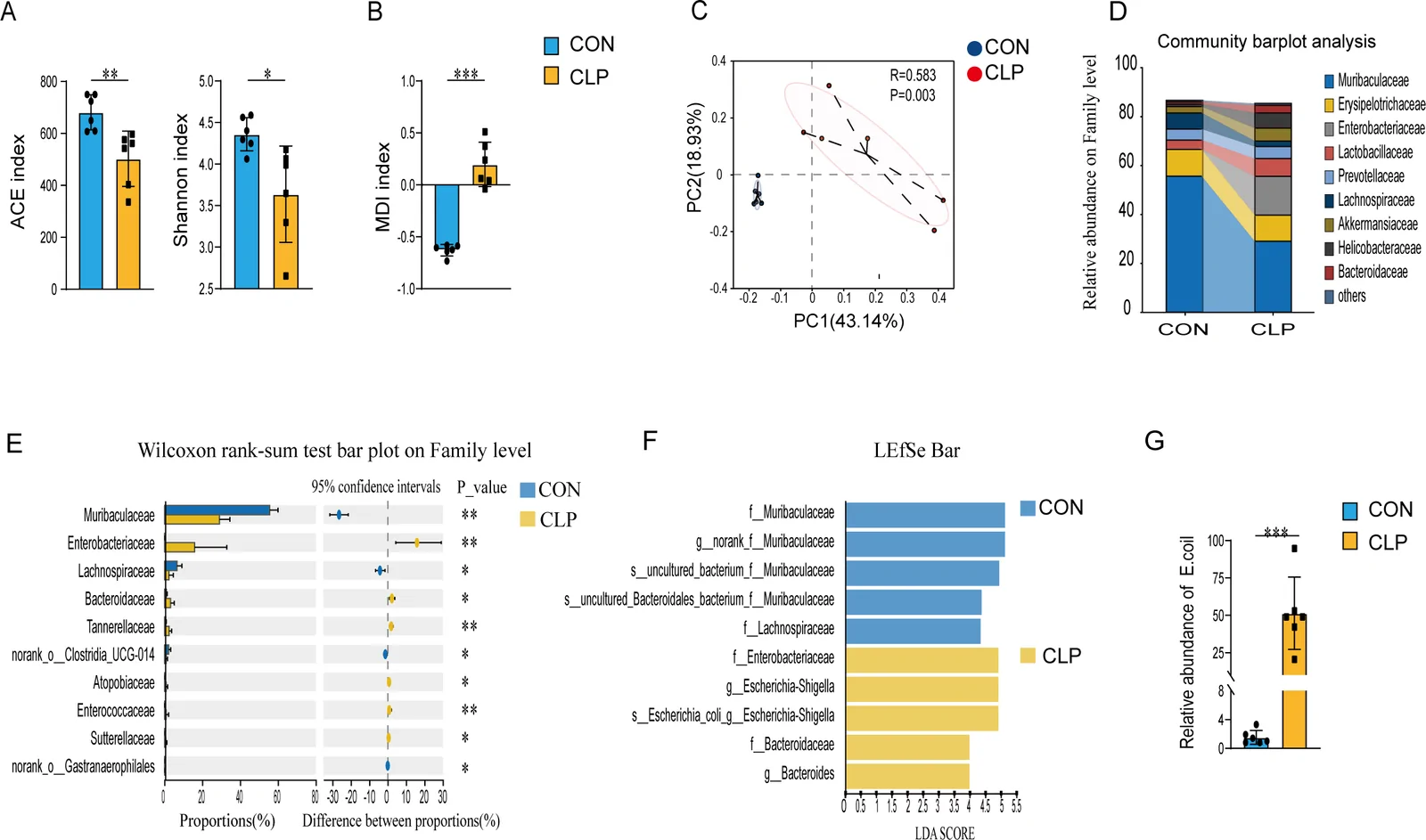
Gut microbiota dysbiosis characterized by *E. coli* expansion in septic mice. Fecal samples were collected from mice on day 2 after CLP or sham (CON) surgery for 16S rRNA gene sequencing analysis. (**A**) Alpha diversity of fecal microbiota assessed by the ACE and Shannon indices. (**B**) Gut microbiota dysbiosis index (MDI). (**C**) PCoA scatter plot of gut microbiota composition displaying beta diversity. (**D**) Relative abundance of the gut microbiota at the family level. (**E**) Significance analysis of the relative abundance at the family level. (**F**) Linear discriminant analysis scores derived from LEfSe analysis of the gut microbiota. (**G**) Comparison of the relative abundance of *E. coli* in feces by qPCR. *P < 0.05, **P < 0.01, and ***P < 0.001. *n* = 6.

To investigate the impact of sepsis on gut microbiota composition, we established a mouse model of sepsis by cecal ligation and puncture (CLP) and collected fecal samples for 16S rRNA sequencing on day two after modeling. The Shannon and Abundance-based Coverage Estimator (ACE) indices were employed to evaluate α-diversity, reflecting species richness and evenness; the microbial dysbiosis index (MDI) was used to assess the extent of microbial dysbiosis; and principal coordinate analysis (PCoA) was applied to determine β-diversity, revealing intergroup differences in community structure. The results demonstrated that, compared with the control group, CLP mice exhibited significantly reduced ACE and Shannon indices (Fig. 1A), along with a markedly increased MDI index (Fig. 1B), indicating a substantial decrease in both richness and diversity of the gut microbiota and the presence of microbial dysbiosis in the sepsis group. PCoA further revealed that the fecal microbial communities of the sepsis and control groups formed distinct clusters (Fig. 1C).

To further characterize the microbial composition and relative abundances in the septic and control groups, as well as to visualize shifts in taxonomic profiles across groups, we generated community bar plots. At the family level, septic mice showed a decreased relative abundance of *Muribaculaceae*, while *Enterobacteriaceae* was expanded (Fig. 1D). Comparative analysis between the two groups confirmed a significant enrichment of *Enterobacteriaceae* in the gut of septic mice (Fig. 1E). Linear discriminant analysis and cladogram results consistently confirmed significant structural divergence in bacterial communities between the two groups. The septic group was predominantly characterized by enrichment of *Enterobacteriaceae*, *Escherichia-Shigella*, and *Escherichia coli* (Fig. 1F). Further quantitative PCR (qPCR) analysis demonstrated a marked expansion of *E. coli* in the intestines of septic mice compared with sham-operated controls (Fig. 1G). These findings suggest that sepsis induces gut microbiota dysbiosis accompanied by substantial overgrowth of *E. coli*.

### Aggravation of CLP-induced neuroinflammation by gut microbiota dysbiosis

**Fig 2:**
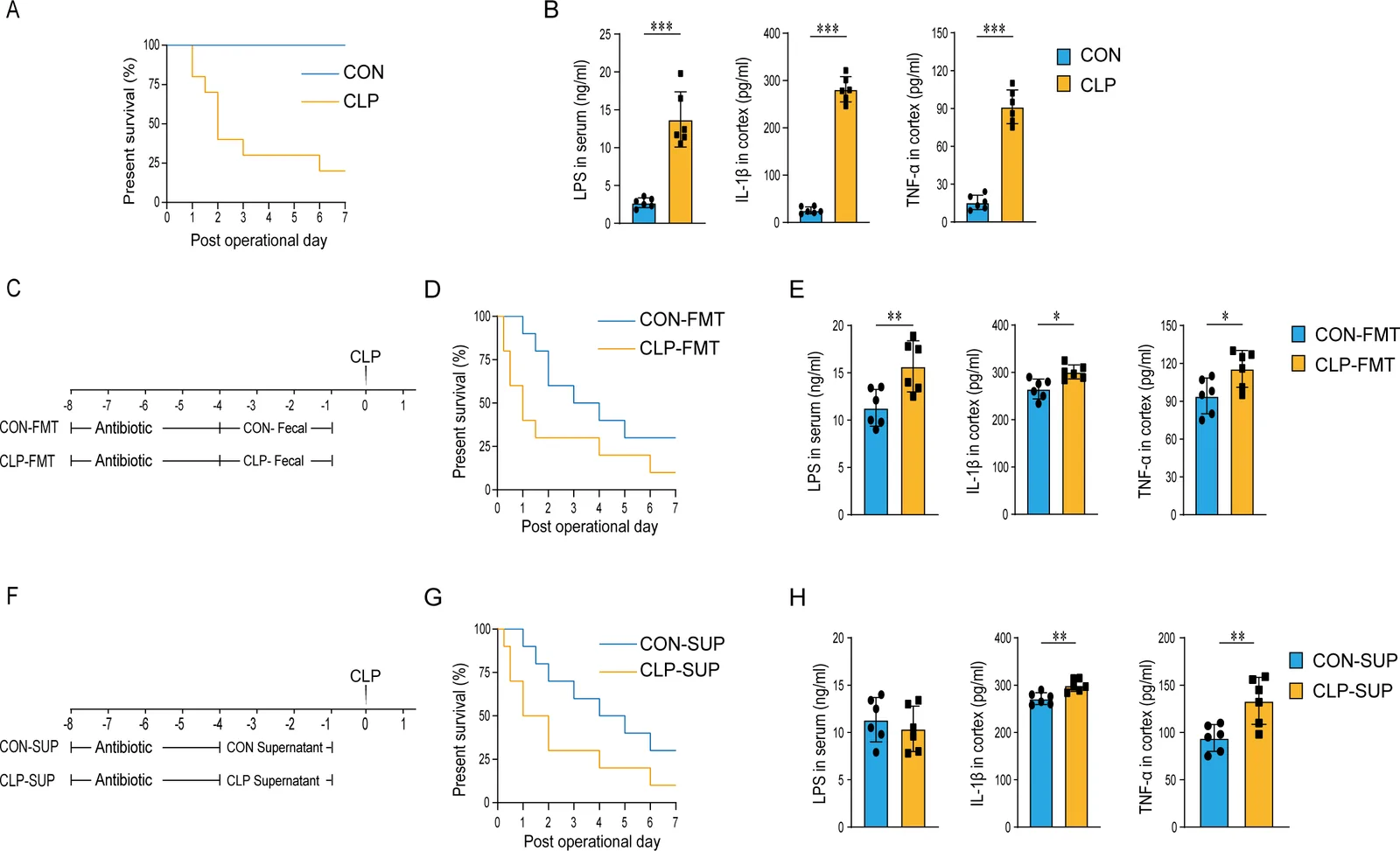
Aggravation of CLP-induced neuroinflammation by gut microbiota dysbiosis. (**A**) Survival observation of mice within 7 days after CLP or sham surgery. (**B**) Serum LPS levels, cortical IL-1β levels, and TNF-α levels in mice. (**C–E**) Mice received daily antibiotics for 5 days, followed by daily fecal microbiota transplantation (FMT) from sham-operated (CON-FMT) or CLP-operated (CLP-FMT) donors for 3 days. (**C**) Schematic diagram of the FMT procedure. (**D**) Survival curves. (**E**) Serum LPS, cortical IL-1β, and TNF-α levels. (**F–H**) Mice received daily antibiotics for 5 days, followed by daily transplantation of sterile fecal supernatant from sham-operated (CON-SUP) or CLP-operated (CLP-SUP) donors for 3 days. (**F**) Schematic diagram of the supernatant transplantation procedure. (**G**) Survival curves. (**H**) Serum LPS, cortical IL-1β, and TNF-α levels. *P < 0.05, **P < 0.01, and ***P < 0.001. *n* = 6–10.

Having observed that sepsis induces significant alterations in the gut microbiome, we explored whether such changes contribute to neuroinflammatory responses. We first evaluated the impact of sepsis on mouse survival and neuroinflammation. As shown in Fig. 2A and B, CLP mice exhibited increased mortality compared with sham-operated controls, accompanied by significantly elevated levels of serum lipopolysaccharide (LPS) and cerebral cortical IL-1β and TNF-α, suggesting that sepsis exacerbates central inflammatory responses. Next, to investigate whether sepsis-induced neuroinflammation can be transferred via the gut microbiota, we performed a fecal microbiota transplantation (FMT) experiment (Fig. 2C). Mice underwent a 5-day antibiotic pretreatment to deplete the resident gut microbiota, followed by FMT from either sham-operated (CON-FMT) or CLP-operated (CLP-FMT) donor mice (Fig. 2C). Survival analysis revealed that the 7-day survival rates after CLP modeling in the CON-FMT and CLP-FMT groups were 30% and 10%, respectively (Fig. 2D). Moreover, compared with the control group, mice transplanted with septic microbiota exhibited significantly elevated levels of serum LPS and cerebral cortical IL-1β and TNF-α (Fig. 2E). These findings suggest that colonization with sepsis-associated gut dysbiosis contributes to both worse survival outcomes and exacerbated neuroinflammatory responses in the CLP model.

**Fig 3:**
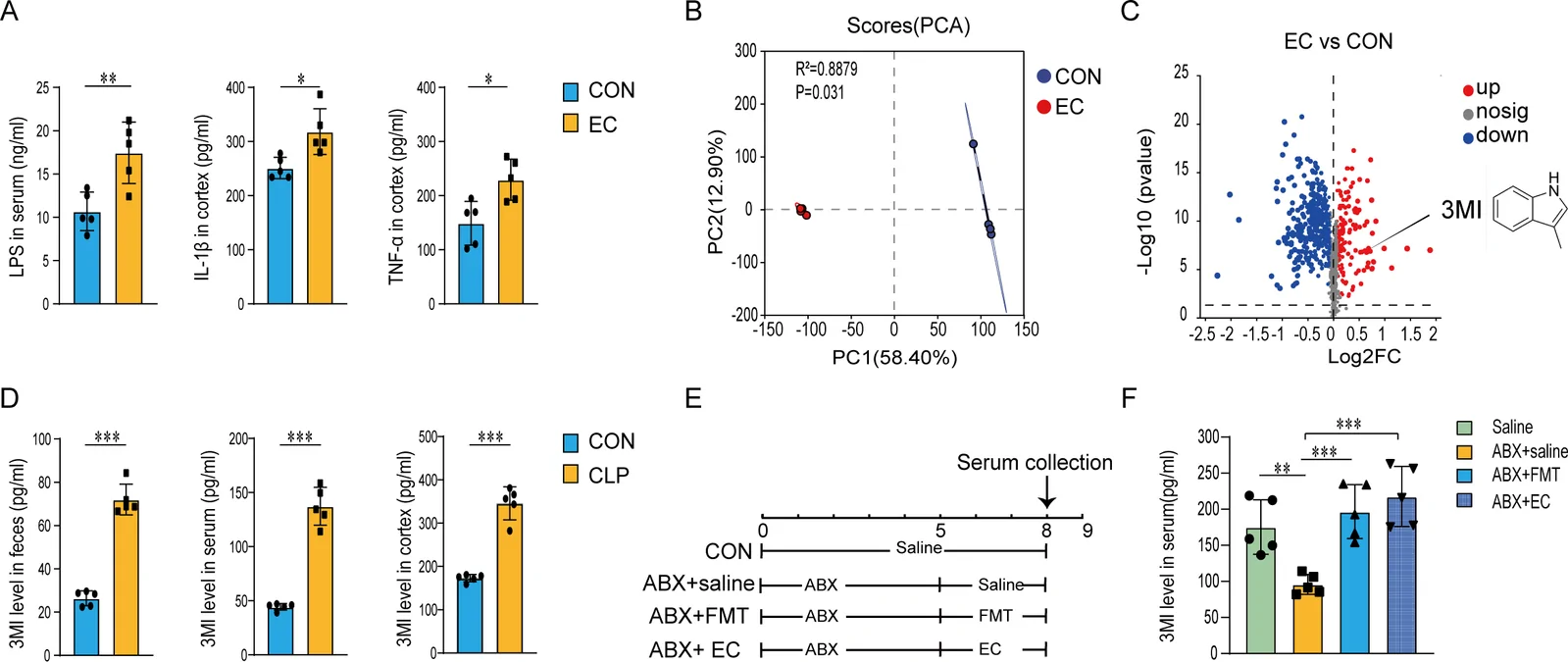
Gut *E. coli* expansion drives serum 3MI enrichment. (**A**) Mice were pretreated with an antibiotic cocktail, followed by daily oral gavage with either an *E. coli* suspension or saline for three consecutive days prior to CLP induction. Serum LPS, cortical IL-1β, and TNF-α levels. (**B and C**) Untargeted metabolomic analysis of *E. coli* culture supernatant versus blank medium supernatant. (**B**) PCA score plot of the supernatant metabolome. (**C**) Volcano plot of differentially abundant metabolites. (**D**) Levels of 3MI in feces and serum from CLP and sham-operated mice. (**E**) Schematic of the bacterial intervention experimental design and procedure. (**F**) Serum 3MI levels in mice after gut microbiota depletion and reconstitution. Mice were treated with antibiotics for 5 days, followed by oral gavage with sterile saline, fecal supernatant from septic mice, or *E. coli* suspension for 3 days. Serum 3MI levels were measured on day 8. *P < 0.05, **P < 0.01, and ***P < 0.001. *n* = 4–5.

Subsequently, we investigated whether gut dysbiosis contributes to neuroinflammation via its metabolites. A fecal microbe-free supernatant transfer experiment was conducted. After a 5-day antibiotic pretreatment, the mice received an oral gavage of sterile fecal supernatants derived from either control or septic donors daily for three consecutive days (Fig. 3F). Interestingly, compared with the control group, the fecal sterile supernatant from septic mice did not further elevate serum LPS levels, yet it did accelerate mortality and aggravate cortical inflammation in CLP model mice (Fig. 3G and H). These data indicate that following gut microbiota dysbiosis induced by sepsis, an abnormally enriched microbial metabolite participates in the regulation of neuroinflammation.

### Gut *E. coli* expansion drives serum 3MI enrichment

To further investigate whether the metabolites regulating neuroinflammation originate from the expansion of gut *E. coli*, we first isolated and cultured this strain from fecal samples of septic mice and conducted mono-colonization experiments. Pseudo germ-free mice colonized with *E. coli* showed higher levels of serum LPS and cerebral cortical IL-1β and TNF-α at 24 h after CLP modeling than controls (Fig. 3A). Next, we performed untargeted metabolomic analysis on the culture supernatant of the *E. coli.* Principal component analysis revealed a clear separation between the metabolic profiles of the *E. coli* culture group and the blank Luria-Bertani (LB) medium control group (Fig. 3B), indicating significant differences in metabolite composition between the two groups. Further analysis revealed that the *E. coli* supernatant was enriched with 3-methylindole (3MI) (Fig. 3C), a tryptophan derivative reported to be associated with interstitial pneumonia and olfactory dysfunction (23, 24). Given these reported activities, 3MI was selected for further investigation. Moreover, 3MI levels were significantly elevated in the feces, serum, and cerebral cortex of CLP mice compared with sham-operated controls (Fig. 3D).

Given the observed differences in serum 3MI concentrations between septic and control mice, we wondered whether serum 3MI levels are regulated by gut *E. coli*. Mice were treated with antibiotics for 5 days to deplete the gut microbiota, followed by oral gavage with sterile saline, fecal supernatant from septic mice, or *E. coli* suspension for 3 days to reconstitute the gut microbiota. Serum 3MI levels were measured on day 8 (Fig. 3E). The results showed that serum 3MI levels were significantly reduced after antibiotic treatment, whereas they were restored following oral gavage with fecal samples from septic mice or *E. coli* (Fig. 3F). Our data clearly demonstrate that serum 3MI levels are regulated by intestinal *E. coli*.

### 3MI synergizes with LPS to exacerbate neuroinflammation

**Fig 4:**
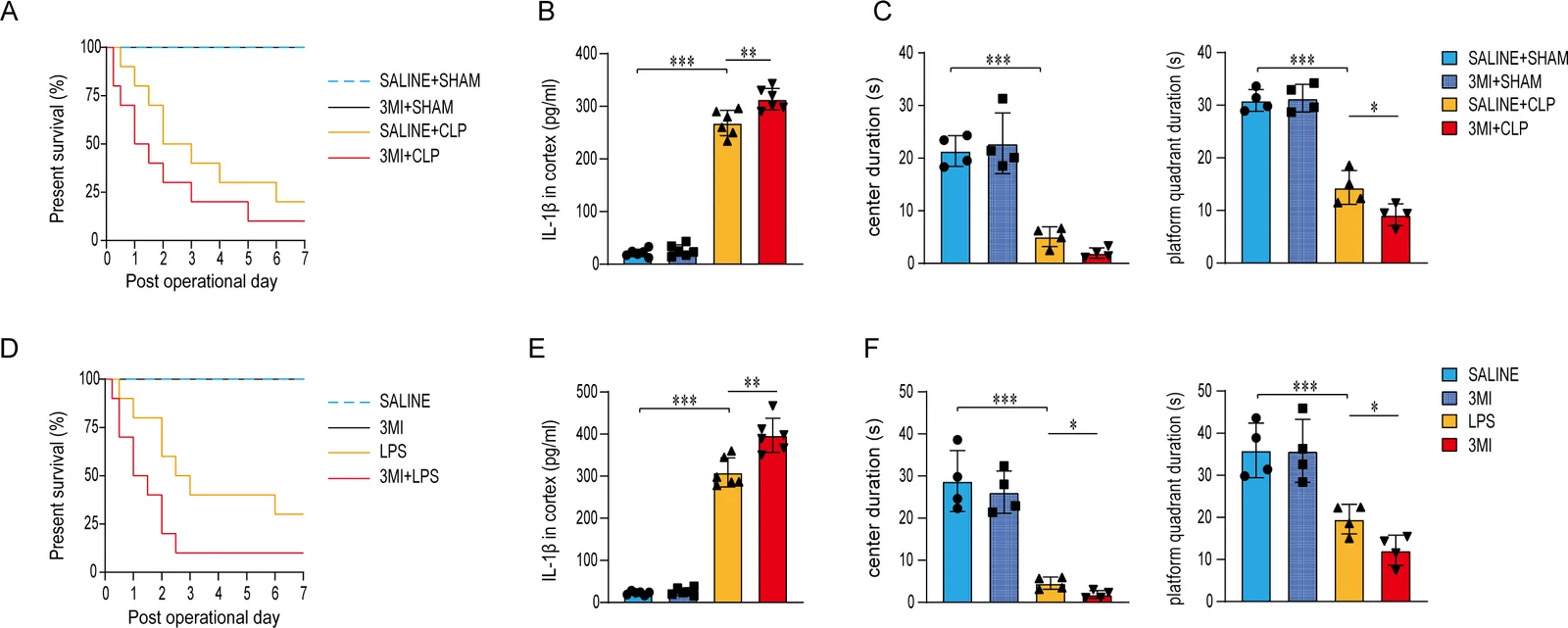
3MI synergizes with LPS to exacerbate neuroinflammation. (**A–C**) Effects of 3MI in the CLP-induced sepsis model. (**A**) Survival curves. (**B**) Cerebral cortical IL-1β levels. (**C**) Behavioral performance, including time spent in the center zone in the open field test and time spent in the target quadrant in the Morris water maze probe trial. (**D–F**) Effects of 3MI in the LPS-induced sepsis model. (**D**) Survival curves. (**E**) Cerebral cortical IL-1β levels. (**F**) Behavioral performance as described in panel **C**. *P < 0.05, **P < 0.01, and ***P < 0.001. *n* = 6–10.

Next, to investigate whether 3MI possesses the potential to induce neuroinflammation, we conducted an *in vivo* administration experiment of 3MI. The survival of mice in each group is shown in Fig. 4A. Regarding the inflammatory response, compared with the CLP + saline group, 3MI intervention significantly increased cerebral cortical IL-1β levels in CLP mice, whereas it had no such effect in the sham-operated group (Fig. 4B). In behavioral tests, mice treated with 3MI exhibited a significant decrease in the duration spent in the center zone during the open field test and in the target quadrant during the probe trial on day 6 of the Morris water maze test, relative to the saline-treated group, with no such alterations observed in sham-operated mice. These findings suggest that 3MI exacerbates brain dysfunction in septic mice (Fig. 4C).

Notably, no such adverse effects were observed in sham-operated mice treated with 3MI. We hypothesized that 3MI-induced exacerbation of inflammation might be associated with LPS. Accordingly, we further assessed the effect of 3MI in an LPS-induced sepsis mouse model. Survival curves for each group are presented in Fig. 4D. In addition, compared with the control group, LPS stimulation significantly increased cerebral cortical IL-1β levels, and co-administration of 3MI further enhanced this effect, whereas 3MI alone had no significant effect on healthy mice (Fig. 4E). In behavioral tests, we observed that 3MI exacerbated LPS-induced brain dysfunction (Fig. 4F). The above findings indicate that 3MI does not directly trigger neuroinflammation but acts in synergy with LPS to aggravate the inflammatory response.

### 3MI enhances NLRP3 inflammasome activation by reducing its ubiquitination

**Fig 5:**
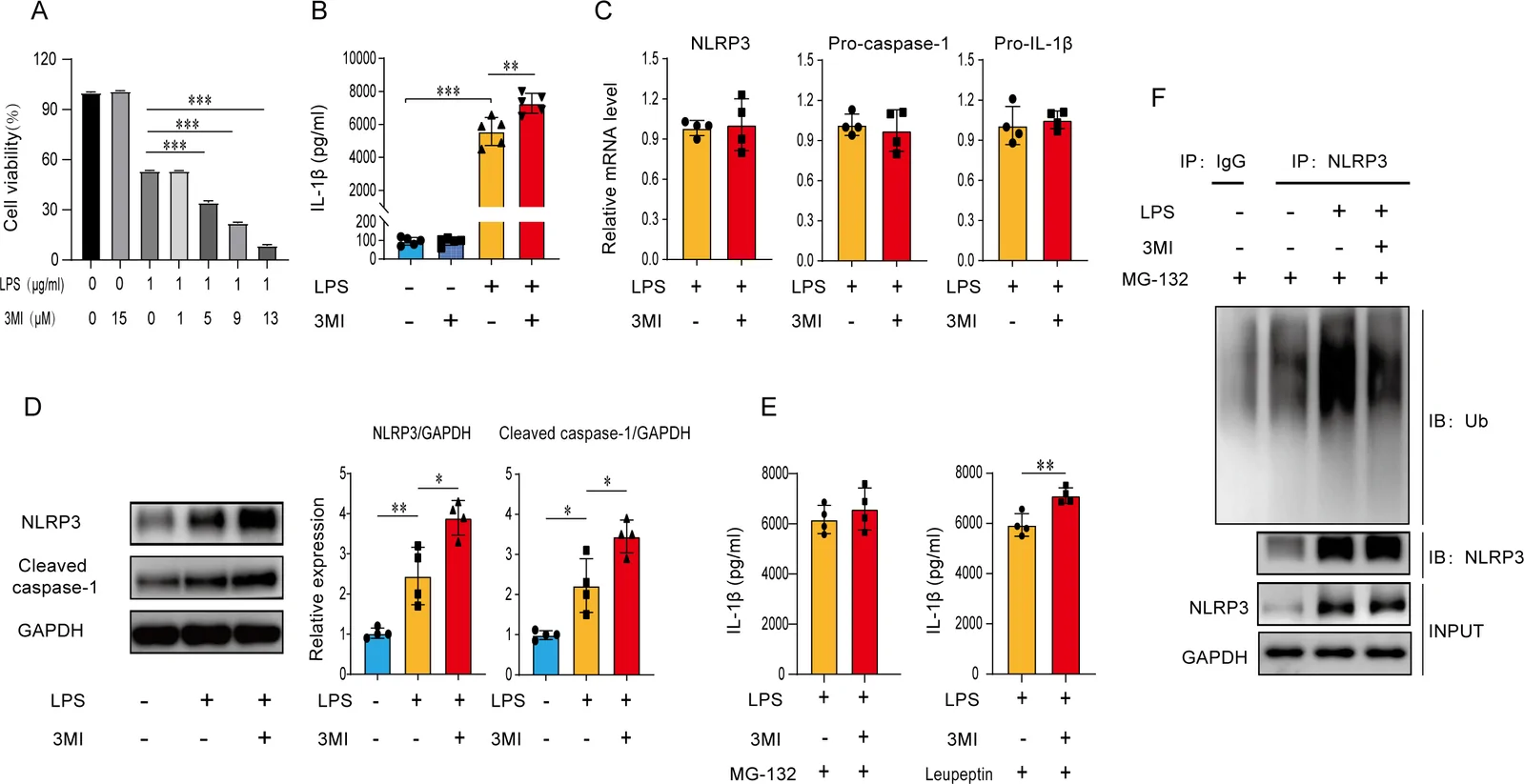
3MI enhances NLRP3 inflammasome activation by reducing its ubiquitination. Mouse primary microglia were pretreated with 5 μM 3MI for 1 hour and then stimulated with 1 mg/mL LPS for 6 hours. (**A**) Cell viability was assessed by CCK-8 assay. (**B**) IL‑1β levels in the supernatant of primary microglia. (**C**) Relative mRNA expression of NLRP3, caspase‑1, and IL‑1β. (**D**) Western blot analysis with quantification of NLRP3 and cleaved caspase‑1 protein levels. (**E**) Effect of 3MI on IL‑1β levels in the culture supernatant of LPS‑stimulated cells in the presence of the proteasome inhibitor MG‑132 or the lysosome inhibitor leupeptin. (**F**) Ubiquitination level of NLRP3 detected by co‑immunoprecipitation (using an anti‑NLRP3 antibody) and Western blotting. *P < 0.05, **P < 0.01, and ***P < 0.001. *n* = 4–6.

Activation of the NLRP3 inflammasome in microglia is a key molecular mechanism of neuroinflammation in SAE (20, 25), yet the effect of 3MI on the NLRP3 inflammasome remains unclear. Thus, we wondered whether 3MI exacerbates neuroinflammation by synergizing with LPS to activate the NLRP3 inflammasome in these cells. Cell Counting Kit-8 (CCK-8) assays showed that 3MI alone at 15 μM did not affect microglial viability. In the presence of LPS, 3MI at 5, 9, and 13 μM enhanced LPS-induced cytotoxicity in a concentration-dependent manner, whereas 1 μM showed no significant effect (Fig. 5A). A concentration of 5 μM was selected for subsequent experiments. Consistent with the *in vivo* findings, *in vitro* studies revealed that treatment with 3MI alone did not significantly increase IL-1β secretion in primary microglia, whereas its co-treatment with LPS markedly enhanced IL-1β levels compared with LPS treatment alone (Fig. 5B). Notably, in LPS-stimulated microglia, 3MI treatment did not significantly affect the mRNA levels of NLRP3, caspase-1, or IL-1β (Fig. 5C) but upregulated the protein levels of NLRP3 and cleaved caspase-1 (Fig. 5D), suggesting that 3MI may be involved in the post-translational modification of NLRP3.

Subsequently, we examined whether 3MI affects the ubiquitination of NLRP3. Primary microglia were treated with 3MI, LPS, the proteasome inhibitor MG-132, or the lysosome inhibitor leupeptin. The results showed that MG-132 abolished the enhancing effect of 3MI on LPS-induced IL-1β secretion, whereas leupeptin had no significant effect (Fig. 5E). Further co-immunoprecipitation analysis revealed that MG-132 treatment abolished the 3MI-induced upregulation of NLRP3 protein levels, and that 3MI reduced the ubiquitination level of NLRP3 (Fig. 5F). These findings suggest that 3MI may enhance NLRP3 protein stability and inflammasome activity by inhibiting its ubiquitination-mediated degradation.

## DISCUSSION

This study demonstrates that expansion of gut *E. coli* is a key factor exacerbating neuroinflammatory responses in CLP-induced septic mice. The amplification of intestinal *E. coli* leads to the enrichment of lipopolysaccharide (LPS) and another pro-neuroinflammatory metabolite—3MI. The potential mechanism underlying the pro-neuroinflammatory effect of 3-MI may involve the reduction of NLRP3 ubiquitination levels in microglia, thereby synergizing with LPS to activate the NLRP3 inflammasome and promote IL-1β secretion. These findings advance our understanding of the regulatory role of the gut microbiota in sepsis-associated organ injury and suggest that gut microbiota-derived 3-MI may act as a mediator in neuroinflammation.

There is considerable interindividual variability with respect to SAE susceptibility (1, 26). Numerous studies have noted that susceptibility to sepsis-associated organ injuries, such as liver and kidney damage, can be modulated by the gut microbiota (27, 28). Our previous study indicated that the gut microbiota is associated with susceptibility to SAE (16), and the present study further investigates the potential mechanisms by which the gut microbiota regulates neuroinflammation. In a mouse model of sepsis induced by CLP, we observed significant gut microbiota dysbiosis. This finding is consistent with previous reports (13, 29), further confirming that sepsis disrupts gut microecological homeostasis and promotes the overgrowth of opportunistic pathogens. Of particular note, a marked enrichment of *Enterobacteriaceae* (especially *E. coli*) was detected in septic mice. The overgrowth of intestinal *Enterobacteriaceae*, an independent prognostic risk factor, is associated with an increased risk of intestinal inflammation, cerebral infarction, and mortality (30–32), implying its important regulatory role in systemic organ injury processes.

We next sought to identify the involvement of sepsis-induced gut dysbiosis accompanied by *E. coli* expansion in neuroinflammatory responses. Using FMT, we demonstrated that sepsis-induced gut dysbiosis significantly elevated serum LPS levels and exacerbated central neuroinflammation in CLP mice. Of note, even intervention with sterile fecal supernatant—which itself did not further elevate LPS levels—still aggravated central neuroinflammation, indicating that dysbiotic microbiota may regulate neuroinflammation via metabolites in addition to LPS. Given the marked enrichment of *E. coli* in the intestines of septic mice, we isolated and cultured the strain, and then confirmed through intestinal colonization experiments that it could aggravate neuroinflammation. We subsequently identified 3MI as an enriched metabolite in its supernatant using metabolomic analysis. Considering its demonstrated association with *E. coli* and frequent use in modeling olfactory dysfunction (33, 34), 3MI was selected as a candidate to explore its potential pro-inflammatory role in SAE. Results showed that 3-MI exacerbated neuroinflammation in CLP-induced septic mice, indicating a signaling role in the gut-brain axis.

Notably, 3MI alone did not induce significant neuroinflammatory responses, whereas its co-administration with LPS markedly amplified such responses, in both *in vivo* and *in vitro* settings. This effect appeared to depend on a pre-existing inflammatory microenvironment, such as a state primed by LPS, suggesting that 3MI acts not as an initial inflammatory trigger but rather as an “amplifier” of inflammation. To elucidate the molecular mechanism underlying 3MI-mediated augmentation of inflammation, we examined its effect on the NLRP3 inflammasome, a key mediator of neuroinflammation in SAE (25). We found that 3MI increased NLRP3 protein levels and promoted caspase-1 activation without affecting its transcription. Further experiments revealed that 3MI reduced the ubiquitination level of NLRP3. These data point to a model whereby 3MI may enhance NLRP3 inflammasome activation by lowering its ubiquitination, leading to sustained inflammatory signaling in microglia.

The phenomenon whereby 3MI enhances the LPS-driven response but causes no inflammation alone may be explained by the two-signal activation mechanism of the NLRP3 inflammasome. This mechanism is generally considered to require two sequential signals: a priming signal (such as the transcription of NLRP3 and pro-IL-1β) and an activation signal (such as inflammasome oligomerization) (35, 36). In this study, LPS stimulation likely provided the necessary priming signal. Against this background, 3MI may enhance the stability of NLRP3 protein by decreasing its ubiquitination, thereby further increasing NLRP3 protein levels and ultimately amplifying the strength of the second signal. Since sufficient NLRP3 protein is crucial for inflammasome assembly and activation, and the basal expression level of NLRP3 in microglia is low in the absence of a priming signal (35–37), this may explain why the pro-inflammatory activity of 3MI largely depends on the inflammatory microenvironment established by LPS pre-stimulation. Certainly, the aforementioned regulatory pathways and specific mechanisms require further in-depth validation and clarification in future studies.

Several limitations of this study should be acknowledged. First, while 3MI was identified as a key differential metabolite, the potential contributions of other metabolites and the interference of endogenous 3MI during exogenous administration were not fully examined. Second, we did not independently verify the depletion of *E. coli* after antibiotic treatment. Third, only male mice were used, which limits the generalizability of our findings given the well-documented sex differences in microglial responses during sepsis. Finally, the precise molecular target through which 3MI modulates NLRP3 ubiquitination, whether by inhibiting a specific E3 ubiquitin ligase or by activating a deubiquitinase, remains undefined in the present study. Future work will be required to further investigate the underlying molecular mechanisms and to validate our observations in both sexes.

In conclusion, our study revealed that sepsis-induced gut dysbiosis with *E. coli* expansion enriches the metabolite 3MI, which may synergize with LPS to potentiate NLRP3 inflammasome activation through decreased NLRP3 ubiquitination, thereby exacerbating neuroinflammation and contributing to SAE.

## MATERIALS AND METHODS

### Animal model

Male specific pathogen-free C57BL/6 mice (6 weeks of age) were used in the experiments. The CLP model was used to induce sepsis in mice. In brief, after anesthesia and midline laparotomy, the cecum was ligated and punctured once with a 21-gauge needle. Sham controls underwent surgery without ligation/puncture. All mice were resuscitated with 1 mL saline subcutaneously. In the LPS-induced sepsis model, sepsis was established by intraperitoneal injection of LPS derived from *E. coli* O111:B4 (Sigma-Aldrich, L4391) at a dose of 1 mg/kg, a dosage with reference to our previous study (38). All mice received an equal volume of LPS solution or phosphate-buffered saline (PBS) (10 mL/kg body weight) by intraperitoneal injection. Before modeling (either CLP or LPS challenge), mice in all experimental groups received daily gavage of either 3MI (10 mg/kg in corn oil, Sigma-Aldrich, M51458) or an equal volume of vehicle for five consecutive days. Mice were housed under controlled conditions with free access to food and water. On day 2 after modeling, all mice in the tissue collection cohort (*n* = 6 per group) were euthanized, and brain tissue and serum were collected from each animal. All samples were then stored at −80°C. A separate cohort of mice (*n* = 10 per group) was used for survival analysis, with survival monitored daily for 7 days without tissue collection. The animal experimental groups and intervention conditions are presented in Table 1.

**TABLE 1:** Summary of experimental design and animal allocation

Experiment	Intervention	Survival cohort (*n*)	Tissue collection cohort (*n*)
CLP model	3MI or vehicle gavage for 5 days prior to CLP/sham surgery	10	6
LPS model	3MI or vehicle gavage for 5 days prior to intraperitoneal LPS/PBS injection	10	6
FMT experiment	Antibiotic pretreatment for 5 days, followed by FMT daily for 3 days, then CLP	10	6
Sterile fecal supernatant transfer experiment	Antibiotic pretreatment for 5 days, followed by sterile fecal supernatant daily for 3 days, then CLP	10	6
*E. coli* colonization experiment	Antibiotic pretreatment for 5 days, followed by oral gavage with *E. coli* suspension or vehicle daily for 3 days, then CLP	–^*a*^	5

^*a*^ “–” indicates that there was no survival cohort for that group.

### Primary culture of microglia and treatment

Primary microglia were isolated from the cerebral cortex of neonatal (1 day old) C57BL/6 mice, as described in our previous study (16). Cortical tissues were aseptically collected, minced, and subjected to trypsin digestion (0.125%, 37°C, 10 min) before fetal bovine serum was added to quench the enzyme. The cell suspension was filtered (200-mesh) and centrifuged (1,000 rpm, 5 min). The pellet was resuspended in complete DMEM/F-12 medium containing 10% fetal bovine serum and 1% penicillin-streptomycin, then transferred to poly-L-lysine-coated vessels and cultured at 37°C with 5% CO_2_ for 12–14 days, with medium changed every 2 days. To harvest microglia, cultures were placed in an orbital shaker at 37°C and shaken at 200 rpm for 2 h. The detached cells were collected and centrifuged at 1,000 rpm for 5 min using a Hengnuo 4-20R benchtop centrifuge. The resulting microglial pellet was resuspended in complete medium and seeded onto poly-L-lysine-coated six-well plates at a density of 1 × 10⁶ cells/mL (1 mL per well) for subculturing. For 3MI treatment, purified microglia were cultured for 24 h, first pretreated with 5 μM 3MI (dissolved in dimethyl sulfoxide) for 1 h, and then stimulated with 1 μg/mL LPS for 6 h.

### Isolation, culture, and identification of fecal *E. coli*

*E. coli* was isolated, purified, and identified according to the following procedure. Fecal material from CLP-induced septic mice was collected and resuspended in sterile PBS at a concentration of 0.125 g/mL. After centrifugation, the supernatant was inoculated into MacConkey broth and cultured with shaking at 37°C for 12–16 h for enrichment. The enriched culture was then diluted, spread onto eosin methylene blue agar plates, and incubated at 37°C for 18–24 h. Typical colonies, appearing dark purple to black in the center with a metallic sheen, were selected. A single colony was inoculated into LB broth and cultured to the logarithmic growth phase for subsequent experiments. For molecular identification, a single colony served as the template for PCR amplification using specific primers (listed in Table 2) and a colony-direct PCR kit (Beyotime, D7280S). The primers were adopted from a previous study (39). Sterile water was used as the negative control, and a known positive sample was included as the positive control. The PCR products were analyzed by agarose gel electrophoresis (gel containing CelRed nucleic acid dye, 120 V, 30 min), and the expected bands were visualized under UV light to confirm the isolate as *E. coli*.

**TABLE 2:** Sequence information for qPCR primers

	Forward primer (5′−3′)	Reverse primer (5′−3′)
*NLRP3*	GTGGAGATCCTAGGTTTCTCTG	CAGGATCTCATTCTCTTGGATC
*caspase-1*	CTGGGACCCTCAAGTTTTGC	CCCTCGGAGAAAGATGTTGAAA
*IL-1β*	TGTAATGAAAGACGGCACACC	TCTTCTTTGGGTATTGCTTGG
*GAPDH*	TGAAGGGTGGAGCCAAAAG	AGTCTTCTGGGTGGCAGTGAT
*E. coli uidA*	ATGCCAGTCCAGCGTTTTTGC	AAAGTGTGGGTCAATAATCAGGAAGTG
*16S*	ACTCCTACGGGAGGCAGCAG	GGACTACHVGGGTWTCTAAT

### Fecal microbiota depletion and transplantation experiment

The FMT experiment was performed with modifications based on a previously described method (16). Recipient mice were randomly assigned to two groups: control donor FMT group and septic donor FMT group. To construct pseudo-germ-free mice, an antibiotic cocktail (200 mg/kg neomycin sulfate, 200 mg/kg metronidazole, 200 mg/kg ampicillin, and 100 mg/kg vancomycin) was administered via oral gavage once daily for five consecutive days to deplete the native gut microbiota. Subsequently, fecal material from donor mice (either healthy controls or septic mice) was collected and resuspended in sterile saline at a concentration of 0.125 g/mL. Recipient mice then received 0.15 mL of this fecal microbiota suspension by oral gavage once daily for three consecutive days, followed by CLP. For survival analysis, mice were monitored daily for 7 days; a separate cohort (*n* = 6 per group) was euthanized at 12 h post-CLP for collection of brain tissue and serum.

### Sterile fecal supernatant transfer experiment

For the preparation of sterile fecal supernatant, fecal material from donor mice was resuspended in sterile saline at 0.125 g/mL, vortexed, centrifuged at 1,000 rpm for 5 min at 4°C, and filtered through a 0.2-μm sterile filter. Pseudo-germ-free mice received 0.15 mL of sterile supernatant (from control or septic donors) by oral gavage once daily for 3 days, followed by CLP. The same sample size and tissue collection scheme were applied as described for the FMT experiment.

### *E. coli* colonization experiment

Pseudo-germ-free mice received 0.2 mL of *E. coli* suspension (1 × 10^6^ CFU/mL in sterile PBS) or an equal volume of sterile PBS by oral gavage once daily for three consecutive days, followed by CLP. Mice were euthanized at 12 h post-CLP for tissue collection.

### Fecal microbiota composition analysis

Total microbial DNA was extracted from fecal samples with the Mag-Bind Soil DNA Kit (Omega Bio-Tek, USA) per the manufacturer’s instructions. The V3-V4 hypervariable region of the bacterial 16S rRNA gene was amplified by PCR following an established protocol (40). Purified amplicons were pooled in equimolar amounts and paired-end sequenced (2 × 300 bp) on an Illumina MiSeq PE300 platform (Illumina, USA) at Majorbio (Shanghai, China). Raw reads were quality-filtered using fastp (version 0.20.0) and assembled using FLASH (version 1.2.11). Reads with an average quality score <20 over a 50-bp sliding window were truncated, and those <50 bp after trimming or containing ambiguous bases were discarded. Paired-end reads were merged with a minimum overlap of 10 bp and a maximum mismatch ratio of 0.2 in the overlap region. Sequences were demultiplexed allowing 0 mismatches for barcodes and up to two mismatches for primers. To minimize the effects of sequencing depth, all samples were rarefied to 34,044 reads per sample, with an average Good’s coverage of 99.9%. Operational taxonomic units (OTUs) were clustered using UPARSE (version 11) at 97% similarity, with chimeras removed during clustering. Taxonomic assignment of OTUs was performed using the RDP classifier (version 11.5) against the SILVA 16S rRNA gene database (release 138.2), with a confidence threshold of 70%. Community composition was analyzed at various taxonomic levels. Subsequent bioinformatic analyses were carried out in Mothur and QIIME2.0. Alpha diversity was calculated with the Shannon, Simpson, Chao, and ACE indices; beta diversity was evaluated by principal coordinate analysis based on Bray-Curtis distances. Taxonomic composition was compared between groups, and the LEfSe Galaxy module was applied to identify differentially abundant taxa and assess biological consistency.

### CCK-8 assay

To evaluate the effect of 3MI on microglial viability, primary microglia were seeded onto 96-well plates at a density of 1 × 10⁴ cells per well. To assess the safety of 3MI, cells were treated with 15 μM 3MI for 24 h. To determine the synergistic effect of 3MI with LPS, cells were treated with various concentrations of 3MI (1, 5, 9, and 13 μM) in the presence of LPS (1 μg/mL) for 24 h. Cell viability was measured using a CCK-8 assay (GLPBIO, GK10001). Briefly, 10 μL of CCK-8 solution was added to each well and incubated for 2 h at 37°C. Absorbance at 450 nm was measured using a microplate reader.

### Western blot analysis

Total protein was isolated from cortical tissue or cells with a commercial extraction kit (Best Bio, BB-3101-100T), and its concentration was measured using a BCA protein assay kit (Thermo Scientific). Aliquots containing 40 µg (tissue) or 25 µg (microglial cells) of protein were heat-denatured, separated by electrophoresis on 12% polyacrylamide gels, and electro transferred to PVDF membranes. The membranes were blocked and then incubated with specific primary antibodies: NLRP3 (Abcam, ab263899), caspase-1 p20 (Affinity, AF4005), and GAPDH (Abcam, ab181602). After washing, a horseradish peroxidase-conjugated secondary antibody (Cell Signaling Technology, 7074S) was applied. Immunoreactive bands were visualized via chemiluminescence (Millipore, WBKLS0500) using an Image Quant LAS500 imager (GE Healthcare Bio-Sciences AB) and quantified with ImageJ software. Protein expression levels were normalized to GAPDH.

### Enzyme-linked immunosorbent assay

Cerebral cortex protein extracts, serum, and cell supernatants were collected. The levels of TNF-α and IL-1β were measured by enzyme-linked immunosorbent assay (ELISA) kit (Cusabio), according to the manufacturer’s instructions. Serum LPS levels were measured using a commercial ELISA kit (Cusabio) according to the manufacturer’s instructions. Levels of 3MI in feces and serum were measured by ELISA kit (MEIMIAN). The levels of inflammatory factors in the tissues were corrected accordingly to the total protein concentration in the extracts.

### qPCR analysis

Quantitative PCR analyses were performed for two distinct applications. For gene expression analysis, total RNA was extracted from tissue homogenates using the RNAeasy Plus Animal RNA Isolation Kit with Spin Column (Beyotime, R0032), reverse transcribed into cDNA using the BeyoRT II cDNA First-Strand Synthesis Kit (RNase H-) (Beyotime, D7168M), and amplified using the BeyoFast SYBR Green qPCR Mix (Beyotime, D7260) on a Biometra TAdvanced Twin 48G system (Analytik Jena, Germany). Expression levels were normalized to the housekeeping gene Gapdh and analyzed via the 2⁻^ΔΔCt^ method. For microbial quantification, genomic DNA was isolated from fecal samples using the Stool Genomic DNA Purification Kit with Spin Column (UElandy, M7426M). The relative abundance of *E. coli* was determined by qPCR using species-specific primers targeting the uidA gene, with the bacterial 16S rRNA gene serving as an internal reference to normalize for total bacterial load. The primers were adopted from previously published studies (41–44). All primer sequences are provided in Table 2.

### Co-immunoprecipitation

Microglial cells were lysed on ice for 30 min in a buffer containing 50 mM Tris (pH 7.8), 150 mM NaCl, 1% (v/v) Nonidet-P40, 0.1% SDS, 10% (v/v) glycerol, a protease inhibitor cocktail, and a deubiquitinase inhibitor. After centrifugation at 12,000 × *g* for 15 min at 4°C, the supernatant was incubated overnight at 4°C with magnetic beads conjugated to an anti-NLRP3 antibody (Abcam, ab263899). The beads were washed, and bound proteins were eluted in SDS-PAGE loading buffer and heat-denatured. The eluted samples were then resolved by SDS-PAGE and transferred to a PVDF membrane. The membrane was probed with an anti-ubiquitin antibody to assess the ubiquitination status of NLRP3.

### Open field test

To assess anxiety-like behavior and motor function, mice that survived for 36 h after CLP were tested in the open field. Each mouse was gently positioned at the center of a square arena (60 × 60 × 60 cm) and permitted to explore freely for 5 min. Activity trajectories were captured using a video tracking system. Following each session, the arena was wiped with 75% alcohol to remove residual odors.

### Morris water maze test

To assess spatial learning memory, surviving septic mice were tested in the Morris water maze 7 days after CLP. Experiments were performed in a circular pool (diameter 1.2 m) containing water at 25.0 ± 1.0 °C (depth 25 cm), with visual cues present. The pool was divided into four quadrants, one containing a submerged platform (9 cm diameter, 1 cm below the surface). On day 1, mice were gently introduced to the pool for a 120-s habituation swim. From days 2 to 5, each mouse completed one trial per day from each quadrant (randomized order), with a 120-s cutoff to find the platform; those failing were manually guided to and kept on it for 60 s. On day 6, a 120-s probe trial was conducted with the platform removed. All sessions were video-tracked to record movement paths and latencies.

### Metabolomics analysis

Liquid chromatography-mass spectrometry was performed on an AB Sciex TripleTOF 5600TM mass spectrometry system (AB SCIEX, USA) for nontargeted metabolomics analysis, as described elsewhere (16). The raw data were further analyzed using the metabolomics software Progenesis QI (version 3.0, Waters Corporation, Milford, USA) for peak detection and alignment, and a data matrix was generated for bioinformatics analysis. Principal component analysis (PCA) using unsupervised methods was performed to demonstrate repeatability within data groups and variability between groups.

### Statistical analysis

Data are expressed as mean ± standard deviation. Comparisons were performed using Student’s *t*-test or one-way analysis of variance, followed by post-hoc tests where appropriate. A *P*-value < 0.05 was considered statistically significant (*P < 0.05, **P < 0.01, and ***P < 0.001). All statistical analyses were performed using SPSS Statistics (version 26.0, IBM Corp., Armonk, NY, USA).

## Data Availability

The data sets used and analyzed during the current study are available from the authors upon reasonable request, and some have already been included in the paper. Raw sequence data of the microbiota that support the findings in our study have been deposited into NCBI’s Sequence Read Archive under accession number PRJNA1450491.

## References

[1] Gofton TE, Young GB. 2012. Sepsis-associated encephalopathy. Nat Rev Neurol 8:557–566. doi:10.1038/nrneurol.2012.183 22986430

[2] Heming N, Mazeraud A, Verdonk F, Bozza FA, Chrétien F, Sharshar T. 2017. Neuroanatomy of sepsis-associated encephalopathy. Crit Care 21:65. doi:10.1186/s13054-017-1643-z PMC5360026 28320461

[3] Sonneville R, Benghanem S, Jeantin L, de Montmollin E, Doman M, Gaudemer A, Thy M, Timsit J-F. 2023. The spectrum of sepsis-associated encephalopathy: a clinical perspective. Crit Care 27:386. doi:10.1186/s13054-023-04655-8 PMC10552444 37798769

[4] Hong Y, Chen P, Gao J, Lin Y, Chen L, Shang X. 2023. Sepsis-associated encephalopathy: from pathophysiology to clinical management. Int Immunopharmacol 124:110800. doi:10.1016/j.intimp.2023.110800 37619410

[5] Zhang X, Huang K, Wu Z, Ding R, Han J, Zhang Y, Lu Y, Lu Y, Si Y. 2026. Sepsis-associated encephalopathy: unraveling molecular mechanisms, emerging therapeutics, and translational frontiers. Pharmacol Ther 278:108971. doi:10.1016/j.pharmthera.2025.108971 41456741

[6] De Waele JJ. 2024. Importance of timely and adequate source control in sepsis and septic shock. J Intensive Med 4:281–286. doi:10.1016/j.jointm.2024.01.002 PMC11258501 39035625

[7] Park JC, Chang L, Kwon H-K, Im S-H. 2025. Beyond the gut: decoding the gut-immune-brain axis in health and disease. Cell Mol Immunol 22:1287–1312. doi:10.1038/s41423-025-01333-3 PMC12575876 40804450

[8] Loh JS, Mak WQ, Tan LKS, Ng CX, Chan HH, Yeow SH, Foo JB, Ong YS, How CW, Khaw KY. 2024. Microbiota-gut-brain axis and its therapeutic applications in neurodegenerative diseases. Signal Transduct Target Ther 9:37. doi:10.1038/s41392-024-01743-1 PMC10869798 38360862

[9] Ross FC, Patangia D, Grimaud G, Lavelle A, Dempsey EM, Ross RP, Stanton C. 2024. The interplay between diet and the gut microbiome: implications for health and disease. Nat Rev Microbiol 22:671–686. doi:10.1038/s41579-024-01068-4 39009882

[10] Wei L, Peng Y, Mao J, Hu Q. 2025. Adaptive evolution in the mammalian gut microbiota: insights and discoveries. Curr Microbiol 82:525. doi:10.1007/s00284-025-04516-3 41003701

[11] Niu M, Chen P. 2021. Crosstalk between gut microbiota and sepsis. Burns Trauma 9:b36. doi:10.1093/burnst/tkab036 PMC8547143 34712743

[12] Mirji G, Bhat SA, El Sayed M, Reiser SK, Gavara SP, Ye Y, Miyamoto T, Zhang W, Vogel P, Cassel J, Liu Q, Goldman AR, Kossenkov A, Zhang N, Shinde RS. 2026. Aromatic microbial metabolite hippuric acid enhances inflammatory responses in macrophages via TLR-MyD88 signaling and lipid remodeling. Cell Rep 45:116749. doi:10.1016/j.celrep.2025.116749 PMC12884656 41477766

[13] Fang H, Fang M, Wang Y, Zhang H, Li J, Chen J, Wu Q, He L, Xu J, Deng J, Liu M, Deng Y, Chen C. 2022. Indole-3-propionic acid as a potential therapeutic agent for sepsis-induced gut microbiota disturbance. Microbiol Spectr 10:e0012522. doi:10.1128/spectrum.00125-22 PMC9241804 35658593

[14] Yang J, Liang J, Hu N, He N, Liu B, Liu G, Qin Y. 2024. The gut microbiota modulates neuroinflammation in Alzheimer’s disease: elucidating crucial factors and mechanistic underpinnings. CNS Neurosci Ther 30:e70091. doi:10.1111/cns.70091 PMC11512114 39460538

[15] Ran Z, Mu B-R, Wang D-M, Ma Q-H, Lu M-H. 2025. Parkinson’s disease and the microbiota-gut-brain axis: metabolites, mechanisms, and innovative therapeutic strategies targeting the gut microbiota. Mol Neurobiol 62:5273–5296. doi:10.1007/s12035-024-04584-9 39531191

[16] Fang H, Wang Y, Deng J, Zhang H, Wu Q, He L, Xu J, Shao X, Ouyang X, He Z, Zhou Q, Wang H, Deng Y, Chen C. 2022. Sepsis-induced gut dysbiosis mediates the susceptibility to sepsis-associated encephalopathy in mice. mSystems 7:e0139921. doi:10.1128/msystems.01399-21 PMC9239149 35642838

[17] Gao S, Jiang Y, Chen Z, Zhao X, Gu J, Wu H, Liao Y, Sun H, Wang J, Chen W. 2023. Metabolic reprogramming of microglia in sepsis-associated encephalopathy: insights from neuroinflammation. Curr Neuropharmacol 21:1992–2005. doi:10.2174/1570159X21666221216162606 PMC10514522 36529923

[18] Kashif AM, Ouyang Y, Li Y, Pan B. 2025. NETosis and pyroptosis of immune cells in sepsis. J Transl Int Med 13:318–327. doi:10.1515/jtim-2025-0035 PMC12371402 40861069

[19] Castro LVG, Gonçalves-de-Albuquerque CF, Silva AR. 2022. Polarization of microglia and its therapeutic potential in sepsis. Int J Mol Sci 23:4925. doi:10.3390/ijms23094925 PMC9101892 35563317

[20] Xiao D, Wang X, Liang W, Yang Y, Du Y, Liu C, Xu F, Yang Y, Wei M, Yang G. 2025. Convergence of sepsis-associated encephalopathy pathogenesis onto microglia. J Transl Med 23:622. doi:10.1186/s12967-025-06635-8 PMC12131422 40462103

[21] Cook J, Prinz M. 2022. Regulation of microglial physiology by the microbiota. Gut Microbes 14:2125739. doi:10.1080/19490976.2022.2125739 PMC9519021 36151874

[22] Yadav S, Dwivedi A, Tripathi A, Tripathi AK. 2022. Therapeutic potential of short-chain fatty acid production by gut microbiota in neurodegenerative disorders. Nutr Res 106:72–84. doi:10.1016/j.nutres.2022.07.007 36152586

[23] Li S-T, Young T-H, Huang T-W. 2021. Regeneration of olfactory neuroepithelium in 3-methylindole-induced anosmic rats treated with intranasal chitosan. BioMaterials 271:120738. doi:10.1016/j.biomaterials.2021.120738 33711565

[24] Haydock LAJ, Fenton RK, Sergejewich L, Veldhuizen RAW, Smerek D, Ojkic D, Caswell JL. 2023. Bronchopneumonia with interstitial pneumonia in beef feedlot cattle: characterization and laboratory investigation. Vet Pathol 60:214–225. doi:10.1177/03009858221146092 PMC9969494 36625178

[25] Zhong H, Liu T, Shang Y, Huang C, Pan S. 2024. Breaking the vicious cycle: targeting the NLRP3 inflammasome for treating sepsis-associated encephalopathy. Biomed Pharmacother 177:117042. doi:10.1016/j.biopha.2024.117042 39004064

[26] Iacobone E, Bailly-Salin J, Polito A, Friedman D, Stevens RD, Sharshar T. 2009. Sepsis-associated encephalopathy and its differential diagnosis. Crit Care Med 37:S331–S336. doi:10.1097/CCM.0b013e3181b6ed58 20046118

[27] Shahid A, Chambers S, Scott-Thomas A, Bhatia M. 2024. Gut microbiota and liver dysfunction in sepsis: the role of inflammatory mediators and therapeutic approaches. Int J Mol Sci 25:13415. doi:10.3390/ijms252413415 PMC11678143 39769181

[28] Winner K, Chanderraj R, Nuppnau M, He Y, Petouhoff A, Falkowski N, Rnajan P, Woods R, Schaub J, Sjoding MW, Dickson RP. 2025. Anti-anaerobic antibiotics, gut microbiota, and sepsis-associated acute kidney injury. Am J Respir Crit Care Med 211:A2954–A2954. doi:10.1164/ajrccm.2025.211.Abstracts.A2954 PMC13138235 40737346

[29] Yuan Y, Liu S, Ding X, Li Y, Zhang X, Song H, Qi X, Zhang Z, Guo K, Sun T. 2022. Early intestinal microbiota changes in aged and adult mice with sepsis. Front Cell Infect Microbiol 12:1061444. doi:10.3389/fcimb.2022.1061444 PMC9831679 36636721

[30] Xu K, Gao X, Xia G, Chen M, Zeng N, Wang S, You C, Tian X, Di H, Tang W, Li P, Wang H, Zeng X, Tan C, Meng F, Li H, He Y, Zhou H, Yin J. 2021. Rapid gut dysbiosis induced by stroke exacerbates brain infarction in turn. Gut 2021:2020–323263. doi:10.1136/gutjnl-2020-323263 33558272

[31] Lopez LR, Ahn J-H, Alves T, Arthur JC. 2022. Microenvironmental factors that shape bacterial metabolites in inflammatory bowel disease. Front Cell Infect Microbiol 12:934619. doi:10.3389/fcimb.2022.934619 PMC9362432 35959366

[32] Salameh TJ, Roth K, Schultz L, Ma Z, Bonavia AS, Broach JR, Hu B, Howrylak JA. 2023. Gut microbiome dynamics and associations with mortality in critically ill patients. Gut Pathog 15:66. doi:10.1186/s13099-023-00567-8 PMC10731755 38115015

[33] Behrendorff JBYH, Moore CD, Kim K-H, Kim D-H, Smith CA, Johnston WA, Yun C-H, Yost GS, Gillam EMJ. 2012. Directed evolution reveals requisite sequence elements in the functional expression of P450 2F1 in *Escherichia coli*. Chem Res Toxicol 25:1964–1974. doi:10.1021/tx300281g PMC3466110 22901340

[34] Rüzgar H, Seden N, Özdemir O, Bingül B, Kirkik D, Yigit O. 2026. Neuroprotective effects of melatonin on olfactory dysfunction: evidence from a 3-methylindole-induced anosmia model. Acta Otolaryngol 146:670–675. doi:10.1080/00016489.2026.2626964 41677204

[35] Huang Y, Xu W, Zhou R. 2021. NLRP3 inflammasome activation and cell death. Cell Mol Immunol 18:2114–2127. doi:10.1038/s41423-021-00740-6 PMC8429580 34321623

[36] Shin HJ, Kim IS, Kim JK, Jo E-K. 2026. Molecular mechanisms of NLRP3 inflammasome activation. Exp Mol Med 58:650–663. doi:10.1038/s12276-026-01656-9 PMC13049041 41876821

[37] Ji X, Dong J, Zhu W, Zhang S, Chen Y, Liu X, Cao Y, Zhao P, Dong Z. 2025. PIM-1 exacerbates sepsis-associated encephalopathy via promoting microglia NLRP3 inflammasome activation. Free Radic Biol Med 241:469–487. doi:10.1016/j.freeradbiomed.2025.09.055 41038286

[38] Jiang S, Wang H, Zhou Q, Li Q, Liu N, Li Z, Chen C, Deng Y. 2021. Melatonin ameliorates axonal hypomyelination of periventricular white matter by transforming A1 to A2 astrocyte via JAK2/STAT3 pathway in septic neonatal rats. J Inflamm Res 14:5919–5937. doi:10.2147/JIR.S337499 PMC8595063 34803390

[39] Yun Z, Zeng L, Huang W, Wu Q, Fan Y, Zheng S, Peng L, Han J, Huang Y, Zhou H, Chen H. 2018. Detection and categorization of diarrheagenic *Escherichia coli* with auto-microfluidic thin-film chip method. Sci Rep 8:12926. doi:10.1038/s41598-018-30765-3 PMC6110803 30150710

[40] Klindworth A, Pruesse E, Schweer T, Peplies J, Quast C, Horn M, Glöckner FO. 2013. Evaluation of general 16S ribosomal RNA gene PCR primers for classical and next-generation sequencing-based diversity studies. Nucleic Acids Res 41:e1. doi:10.1093/nar/gks808 PMC3592464 22933715

[41] Yuan X, Chen P, Luan X, Yu C, Miao L, Zuo Y, Liu A, Sun T, Di G. 2024. NLRP3 deficiency protects against acetaminophen‑induced liver injury by inhibiting hepatocyte pyroptosis. Mol Med Rep 29:61. doi:10.3892/mmr.2024.13185 PMC10902631 38391117

[42] Qu X-F, Zhai B-Z, Hu W-L, Lou M-H, Chen Y-H, Liu Y-F, Chen J-G, Mei S, You Z-Q, Liu Z, Zhang L-J, Zhang Y-H, Wang Y. 2022. Pyrroloquinoline quinone ameliorates diabetic cardiomyopathy by inhibiting the pyroptosis signaling pathway in C57BL/6 mice and AC16 cells. Eur J Nutr 61:1823–1836. doi:10.1007/s00394-021-02768-w PMC9106599 34997266

[43] Lu Y, Liu M, Guo X, Wang P, Zeng F, Wang H, Tang J, Qin Z, Tao T. 2023. miR-26a-5p alleviates CFA-induced chronic inflammatory hyperalgesia through Wnt5a/CaMKII/NFAT signaling in mice. Cns Neurosci Ther 29:1254–1271. doi:10.1111/cns.14099 PMC10068476 36756710

[44] Xu S, Zhang Y, Ding X, Yang Y, Gao J, Zou N, Lu L, He J. 2025. Intestinal microbiota affects the progression of colorectal cancer by participating in the host intestinal arginine catabolism. Cell Rep 44:115370. doi:10.1016/j.celrep.2025.115370 40022728

